# Increased acid sphingomyelinase levels in pediatric patients with obesity

**DOI:** 10.1038/s41598-022-14687-9

**Published:** 2022-06-29

**Authors:** Chiara Mameli, Carla Carnovale, Federico Ambrogi, Gabriele Infante, Paulina Roux Biejat, Alessandra Napoli, Marco Coazzoli, Valeria Calcaterra, Laura Schneider, Chiara Perazzi, Gianvincenzo Zuccotti, Emilio Clementi, Claudia Moscheni, Cristiana Perrotta

**Affiliations:** 1 Department of Pediatrics, Ospedale dei Bambini V. Buzzi, Via Castelvetro 32, Milan, Italy; 2 Department of Biomedical and Clinical Science L. Sacco, Università Di Milano, Milan, Italy; 3 Laboratory of Medical Statistics and Biometry, ‘Giulio A. Maccacaro’, Department of Clinical Sciences and Community Health, Campus Cascina Rosa, University of Milan, Milan, Italy; 4 Scientific Directorate, IRCCS Policlinico San Donato, San Donato Milanese, Italy; 5 Clinical Epidemiology and Trial Organization Unit, Department of Applied Research and Technological Development, Fondazione IRCCS Istituto Nazionale Tumori, Milan, Italy; 6 Pediatric and Adolescent Unit, Department of Internal Medicine, University of Pavia, 27100 Pavia, Italy; 7 E. Medea Scientific Institute, Bosisio Parini, Italy

**Keywords:** Obesity, Paediatric research

## Abstract

The level of secretory acid sphingomyelinase (S-ASM), a key enzyme in the sphingolipid metabolism, is elevated in a variety of human diseases, including in the serum of obese adults. Alterations in S-ASM were also found to induce morphological changes in erythrocytes. Consequently, the inhibition of S-ASM by functional Inhibitors of ASM (FIASMA) may have broad clinical implications. The purpose of this study was to assess S-ASM activity in pediatric patients with obesity and healthy matched controls, as well as to investigate the erythrocyte morphology using transmission electron microscopy. We recruited 46 obese patients (mean age 11 ± 2.9 years) and 44 controls (mean age 10.8 ± 2.9 years). S-ASM activity was significantly higher (Wilcoxon signed-rank test p-value: 0.004) in obese patients (mean 396.4 ± 49.7 pmol/ml/h) than in controls (mean 373.7 ± 23.1 pmol/ml/h). No evidence of morphological differences in erythrocytes was found between the two populations. We then carried out a case–control study based on the spontaneous reporting system database to compare FIASMAs with NON-FIASMAs in terms of weight gain risk. Children who received FIASMA had a significantly lower frequency of weight gain reports than patients who took NON-FIASMA agents (p < 0.001). Our findings suggest there is an intriguing possibility that S-ASM may play a role in pediatric obesity. This pilot study could serve as the basis for future studies in this interesting field of research.

## Introduction

Obesity is a complex disease that affects children across all age groups worldwide. Obesity is a high risk factor for the development of insulin resistance, type 2 diabetes, and cardiovascular diseases. The molecular changes in obesity that induce these conditions are not completely understood^1^.

Research over the past decade has shed light on the role of alterations in sphingolipid metabolism in obesity’s pathological sequelae^2^. Among bioactive sphingolipids, ceramide, the central constituent of all complex sphingolipids, accumulates under conditions of nutritional overload and triggers metabolic pathways driving insulin resistance, triglyceride production, apoptosis, and fibrosis^2^. The acid sphingomyelinase (ASM) enzyme, encoded by the SMPD1 gene, generates ceramide from the membrane sphingomyelin^3,4^. The enzyme has two forms, which are encoded by the same gene: one located within the lysosome (L-ASM) and the other, the secretory ASM (S-ASM), is released by the cells into the extracellular milieu in response to stress conditions^5,6^. An alteration in both the lysosomal and secretory form has been observed in several human diseases, often related to inflammation^7–13^. Notably, ASM expression and activity were found to be elevated in adipose tissue biopsies of adult obese patients^14^. Secretion by endothelial cells is thought to be the cause of the elevated S-ASM activity in serum observed in patients with inflammatory conditions, including type 2 diabetes^15–17^. In vitro experiments conducted on human endothelial cells revealed that incubation with inflammatory cytokines, such as interleukin-1β and interferon-γ, boosted S-ASM secretion by cells^18^, thus establishing a further link between inflammation and S-ASM.

Exposure to S-ASM induces erythrocyte changes in morphology, deformability, membrane lipid organization, protein–protein interactions, and vesiculation, contributing to an enhanced erythrocyte clearance often associated with inflammation^19^. Erythrocyte dysfunction may occur in the course of obesity and serves as a marker of atherosclerosis, the risk of which is higher in obese children^20–22^.

Studies in mice have demonstrated that the ablation of ASM, obtained by using the ASM knockout mouse model or by administering amitriptyline, an inhibitor of ASM, protects mice fed with a high-fat diet from Nlrp3 inflammasome activation, adipocyte hypertrophy, and weight gain^23,24^. The potential pathological role of ASM in various diseases has prompted studies to identify drugs that can inhibit the enzyme’s activity; several have been described and collectively defined as Functional Inhibitors of ASM (FIASMA)^25–27^. Worth noting is that most of the FIASMAs identified so far are already approved for medical use in humans with different clinical indications (i.e., depression and psychotic disorders) and possess low toxicity^25–27^.

The main objective of this study was to evaluate the activity of S-ASM in the blood serum of a cohort of obese children and adolescents and normal-weight controls, and to identify its possible role in pediatric obesity by identifying characteristics associated with high S-ASM levels, independently of pairing. To this end, we used transmission electron microscopy to also investigate erythrocyte morphological modifications induced by high levels of S-ASM in both populations. Finally, we conducted a nested case–control study based on one of the largest spontaneous reporting databases, *i.e*., the FAERS. We intended to compare FIASMAs with NON-FIASMAs in terms of the risk of reporting anthropometric variations (e.g., weight gain), in a pediatric cohort of patients treated with antipsychotic and antidepressant drugs, the use of which is often associated with an increased risk of weight alteration. Our research hypothesis is based on the probable assumption that antipsychotic and antidepressant agents belonging to the FIASMA class result in a reduced risk of body changes than NON-FIASMA drugs, within the same pediatric cohort of patients.

Consequently, through a multidisciplinary approach, we aim to obtain preliminary innovative results that will shed light on the intriguing possibility that S-ASM may play a pivotal role in the pathogenesis of pediatric obesity and is, therefore, a potential therapeutic target.

## Results

We recruited 46 patients (mean age 11 ± 2.9 years; 19 [41%] males) and 44 healthy controls (mean age 10.8 ± 2.9 years; 17 [38.6%] males), who fulfilled the inclusion criteria. Table 1 gives the baseline characteristics of the patients and controls, including blood pressure and biochemical variables. One patient (2.2%) had hypertension, two patients (4.3%) had hypercholesterolemia, six (13.0%) had hypo-HDL, one (2.2%) had hyper-LDL, and four (8.7%) had hyper-triglycerides.

**Table 1:** Patients' characteristics.

	Patients (n = 46)	Controls (n = 44)	Wald test p-value*
**Age, yrs**			0.372
Mean (SD)	11.0 (2.9)	10.8 (2.9)	
**Gender**			1.000
Male, n (%)	19 (41.3)	17 (38.6)	
**Weight (kg)**			< 0.001
Mean (SD)	59.8 (18.7)	35.2 (13.4)	
**Height (cm)**			< 0.001
Mean (SD)	146.3 (15.1)	139.0 (16.7)	
**Standardized Height**			< 0.001
Mean (SD)	0.5 (1.2)	-0.5 (1.4)	
**BMI (kg/m**^**2**^**)**			< 0.001
Mean (SD)	27.1 (3.7)	17.5 (2.8)	
**SDS BMI**			< 0.001
Mean (SD)	2.7 (0.5)	0.0 (0.9)	
**WC (cm) **2 N/A			< 0.001
Mean (SD)	85.2 (12.8)	61.0 (8.8)	
**Pubertal stages, n (%)**			0.653
Prepubertal	20 (43.5)	24 (54.5)	
Pubertal	8 (17.4)	3 (6.8)	
Postpubertal	7 (15.2)	9 (20.5)	
N/A	11 (23.9)	8 (18.2)	
**SBP (mmHg) ** 6 N/A			0.143
Mean (SD)	106.4 (10.8)	102.9 (10.2)	
**DBP (mmHg)** 6 N/A			0.622
Mean (SD)	66.9 (5.8)	65.8 (7.9)	
**Glycaemia (mg/dl)** 1 N/A 15 N/A			< 0.001
Mean (SD)	82.6 (7.2)	73.4 (8.3)	
**TC (mg/dl)** 11 N/A 30 N/A			0.630
Mean (SD)	165.5 (23.3)	157.6 (39.8)	
**HDL (mg/dl)** 11 N/A 30 N/A			0.115
Mean (SD)	49.7 (13.3)	61.7 (9.6)	
**LDL (mg/dl)** 20 N/A 35 N/A			0.333
Mean (SD)	95.9 (21.0)	103.0 (21.2)	
**TG (mg/dl) **12 N/A 30 N/A			0.246
Mean (SD)	98.0 (68.7)	73.1 (22.8)	
**ALT (mg/dl) **12 N/A 29 N/A			0.307
Mean (SD)	25.6 (12.9)	22.8 (11.6)	

*BMI* body mass index, *DBP* systolic blood pressure, *HDL* high density lipoprotein, *LDL* low-density lipoprotein, *N* Number, *N/A* Not available, *SBP* systolic blood pressure, *TC* total cholesterol, *TG* tryglicerides, *WC* waist circumference * applied to univariable conditional logistic model for matched data.

All patients and healthy controls were non-smokers.

### S-ASM activity

**Figure 1:**
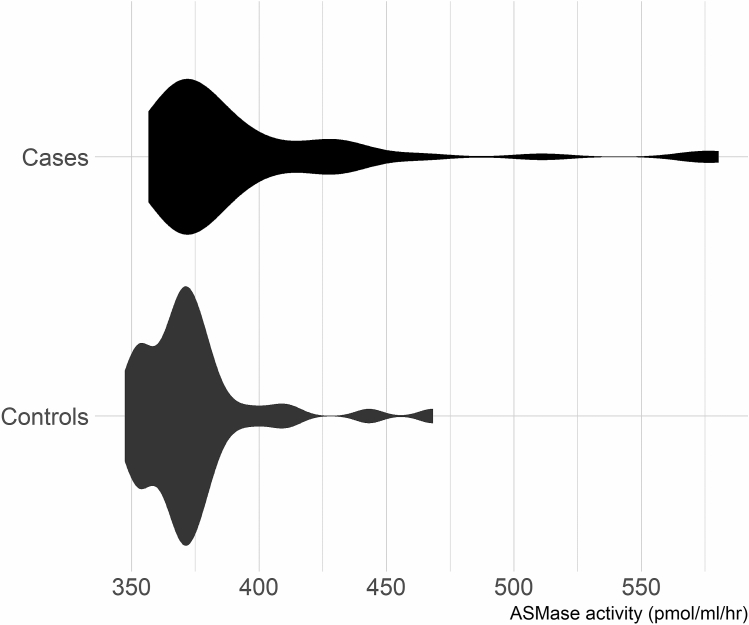
Distribution of the activity of serum acute sphingomyelinase in obese pediatric patients and healthy controls.

S-ASM activity was significantly higher (Wilcoxon-Signed-Rank test p-value: 0.004) in obese pediatric patients (mean 396.4 ± 49.7 pmol/ml/h) than in controls (mean 373.7 ± 23.1 pmol/ml/h). Figure 1 depicts the graphical representation of the different S-ASM distributions between cases and controls.

When a conditional logistic model was applied, an odds ratio (OR) of 1.66 (95% CI 1.13–2.45) was estimated for the 10-unit increment of S-ASM (Wald p-value < 0.001). In other words, we expected to observe an obese subject with a 66% higher probability for each 10 pmol/ml/h.

The only variables that were differently distributed after dividing the subjects by the 3rd quartile of the S-ASM distribution into High- and Low-S-ASM were r sex (Fisher test p-value < 0.01), age (Wilcoxon-Mann–Whitney test p-value < 0.01), height (Wilcoxon-Mann–Whitney test p-value < 0.01), and standardised Body Mass Index (BMI) (Wilcoxon-Mann–Whitney test p-value 0.03). Due to a statistically significant correlation of the subjects’ height with both age (Pearson’s correlation coefficient 0.82, p-value < 0.001) and standardised BMI (Pearson’s correlation coefficient 0.24, p-value < 0.01), height was not included in the set of covariates for an exploratory multivariable logistic regression model with High- vs. Low-S-ASM as the binary outcome. The results of the univariable and multivariable models are reported in Table 2.

**Table 2:** Univariable and multivariable logistic regression model with high- vs low S-ASM.

	Univariable		Multivariable	
	OR (CI 95%)	Wald p-value	OR (CI 95%)	Wald p-value
Sex (Female vs Male)	4.34 (1.33–14.13)	0.015	7.53 (1.86–30.55)	0.005
Age (1-year decrease)	1.41 (1.14–1.74)	0.002	1.44 (1.15–1.82)	0.002
BMI SDS (unit increase)	1.50 (1.06–2.13)	0.023	1.78 (1.16–2.74)	0.008

*BMI* body mass index, *CI* confidence interval, *OR* odds ratio, *S-ASM* secretory acid sphingomyelinase, *SDS* standard deviation score.

The Wald test confirmed all three covariates as statistically significant. Sex was the covariate with the strongest association: females have about an eight-fold higher chance of having high levels of S-ASM than males. An interpretation of the remaining variable’s odds ratio estimates reveals that the likelihood of belonging to the High-S-ASM category increases the younger the subject and the higher the BMI standard deviation score (SDS). Quantitatively, the confidence intervals of each variable’s odds ratios did not include the unit. To better interpret the resulting odds ratios in Table 2, Supplementary Fig. 1 illustrates the predicted log odds for each variable.

### Erythrocyte morphology

Transmission electron microscopy was used to examine the possibility of erythrocyte morphological modifications induced by high levels of S-ASM activity expressed by pediatric patients with obesity compared to healthy controls.

**Figure 2:**
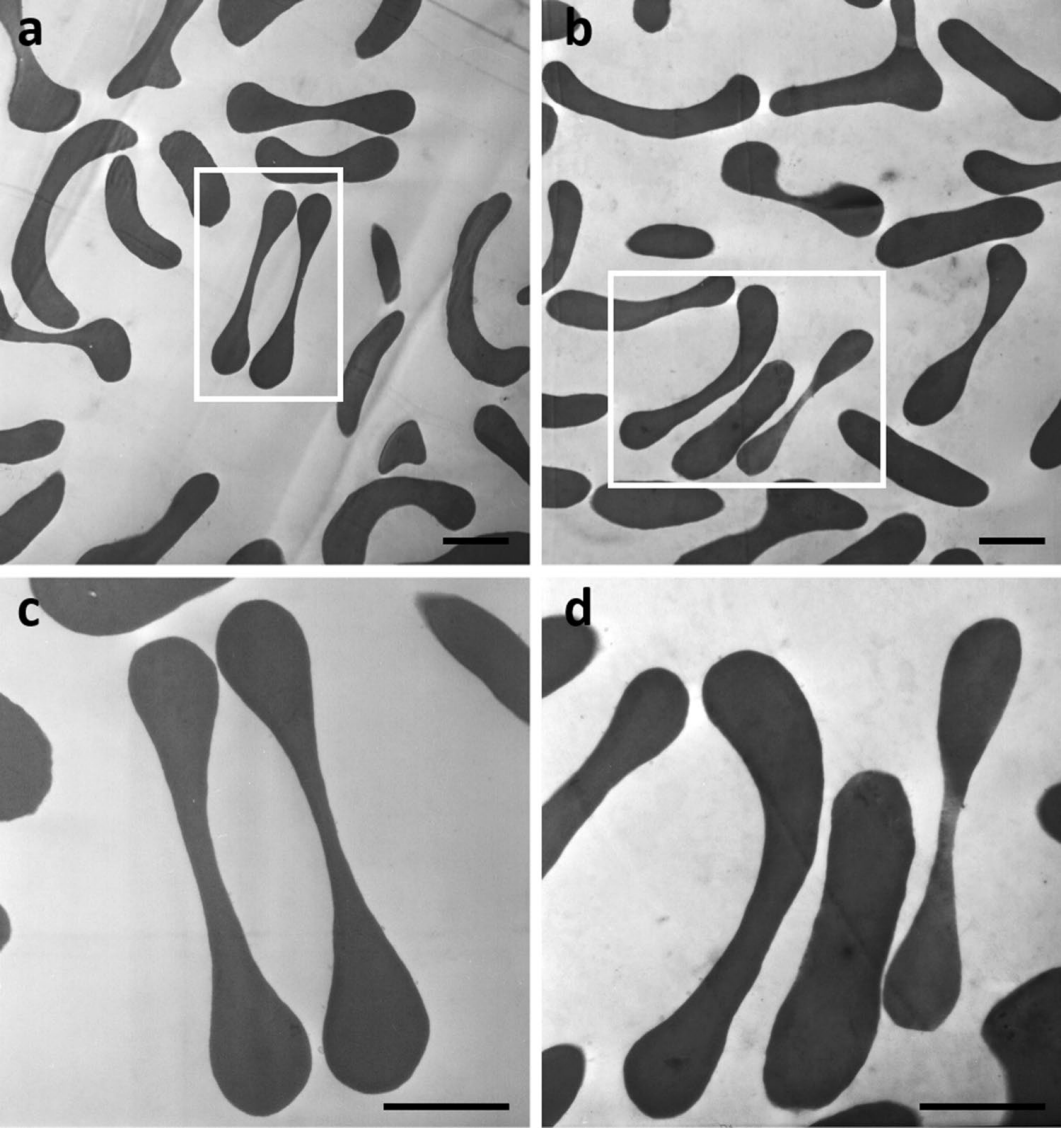
Representative transmission electron microscopy images of obese pediatric patients (**a**–**c**) and healthy controls (**b**–**d**) erythrocytes. In both situations the erythrocytes shape and membranes are well preserved and intracellular membrane vesicles are not detectable. Upper and lower panels scale bar = 1 μm.

No evidence of morphological differences between the erythrocytes of obese and healthy pediatric subjects was shown. Erythrocytes maintain their typical biconcave shape, and membrane irregularities or intracellular membrane vesicles were not observed (Fig. 2).

### Pharmacovigilance analysis

Reports sourced from the FAERS included at least one antidepressant/antipsychotic agent and information on age (5–17 years old) and sex. 11,735 reports were identified following the cleaning procedure (Table 3). Of these, 2,620 (22%) reports were related to body changes (cases); the mean (SD) age of cases was 12 (4) years, with a majority of male patients (n = 2,453; 94%); 51% of children were suffering from attention deficit hyperactivity disorder (ADHD), followed by other psychotic disorders, including bipolar disorder (18%) and autism (13%).

**Table 3:** Characteristics of cases and non-cases included in the pharmacovigilance analysis.

	Cases^a^:Weight gain (n = 2,620)	Non-cases^b^: Other ADRs (n = 9,115)
**Age, yrs**		
Mean (SD)	12(4)	13(3)
**Gender, n (%)**		
Male	2,453(94)	5,347(59)
**Indication use, n (%)**		
ADHD	1,335 (51)	1,491 (16)
Autism	342 (13)	404 (4)
Bipolar Disorder	459 (18)	706 (8)
Depression	259 (10)	1,394 (15)
Schizophrenia	158 (6)	447 (5)
**Concomitants with risk of weight gain, n (%)**		
Yes	23 (1)	356 (4)
**Reporter type, n (%)**		
Consumer	2,411 (92)	4,042 (44)
Lawyer	13 (1)	42 (1)
Physician	89 (3)	2,113 (23)
Pharmacist	10 (0.4)	672 (7)
Health professional	67 (3)	1,844 (20)

*ADHD* Attention deficit hyperactivity disorder, *ADR* adverse drug reactions, *CI* confidence interval, *N* number, *ROR* reporting odds ratio, *SD* standard deviation, *yrs* years.

^a^Pediatric report (5–17 years old) involving at least one antidepressant/antipsychotic agent in the occurrence of weight gain.

^b^Pediatric reports (5–17 years old) involving at least one antidepressant/antipsychotic agent in the occurrence of any type of adverse drug reactions.

Adverse drug reaction reports were most frequently reported by consumers (92%) and healthcare professionals (6.4%). Low percentages of concomitant medications associated with weight gain were used in both groups (1% and 4% for cases and non-cases, respectively).

Children treated with antidepressant and/or antipsychotic agents from the FIASMA class had a lower reported risk of weight gain than patients who were not taking FIASMA medication (Table 4), reaching a statistically significant difference between the two groups (aOR [95% CI] = 0.055 [0.0.038; 0.076]; p < 0.001]).

**Table 4:** Logistic regression analyses showing the reporting risk of weight gain after FIASMA based therapy compared to NON FIASMA (reference group), also according to gender (subgroup analysis).

	Cases (n)	Non cases (n)	ROR (95% CI)	aROR (95% CI)^§^
**FIASMA VS NON FIASMA**				
NON FIASMA (Ref. group)	2,588	7,332	–	–
FIASMA	32	1,783	0.051 (0.035; 0.071)**	0.055 (0.038; 0.076)**
***Subgroup analysis: FIASMA VS NON FIASMA: Male (n* = *7,800)***				
NON FIASMA (Ref. group)	2,441	4,639	–	–
FIASMA	12	708	0.032 (0.017; 0.055)**	0.033 (0.018; 0.056)**
***Subgroup analysis: FIASMA VS NON FIASMA: Female (n* = *3,935)***				
NON FIASMA (Ref. group)	147	2,693	–	–
FIASMA	20	1,075	0.341 (0.206; 0.533)**	0.344 (0.208; 0.538)**

**P < 0.001.

^§^Adjustment for age, concomitant drugs related with the risk of weight gain.

Furthermore, the sub-analysis by gender reported in Table 4 demonstrated that male patients treated with FIASMAs are less prone to weight gain risk (aOR [95% CI] = 0.033 [0.018; 0.056]; p < 0.001] than females (aOR [95% CI] = 0.344 [0.208; 0.538], p < 0.001).

## Discussion

Increased activity of both the lysosomal and secreted isoform of ASM has been observed in several human diseases^5^, supporting the notion that several forms of cellular stress serve as a trigger for enzyme activation. The pathogenic role of these changes is also emerging, given that this dysfunctional activation plays a role in the disease pathogenesis^6^. The role of ASM in pediatric obesity has not yet been assessed.

Using a multidisciplinary approach that combines data from an observational study and a pharmacovigilance analysis, we provide evidence that S-ASM may play a role in childhood obesity.

First, we observed a significant increase in S-ASM activity in the serum of obese Caucasian children and adolescents compared to the healthy control group. To the best of our knowledge, this is the first report that discusses the activity of S-ASM in a pediatric population.

Increased S-ASM activity is consistent with previously published papers demonstrating that dysregulation of sphingolipid metabolism correlates with the pathogenesis of obesity and associated metabolic diseases^28,29^. Several lines of evidence, from in vivo mouse studies and analyses of metabolomics and lipidomics datasets from patients, support a correlation between increased levels of ceramide in tissues and in blood serum in obesity and suggest a mechanistic role played by ceramide, the main product of ASM activity, in the pathophysiology of metabolic diseases^30–33^. Therefore, inhibiting the generation of ceramide may be considered a strategy to prevent obesity-related complications^34,35^.

Recent reports suggest a correlation between ASM hyperactivation and obesity. In an obesity mouse model, hyperinsulinemia and elevated TNF-α levels have been shown to increase ASM expression in adipose tissue^25^.

In diabetic and non-diabetic obese adult patients, ASM expression in adipose tissue was much higher than in lean controls, although activity decreased^14^. Considering the common origin of L-ASM and S-ASM, this discrepancy may be explained by an increase in S-ASM activity in the blood serum. However, this was not evaluated by the article’s authors.

We also demonstrated a strong association between both gender and age and S-ASM activity. Females and younger children are most likely to have high S-ASM levels. To date, the mechanism underlying these associations has not been investigated. However, a gender-specific effect of S-ASM activity was reported in other diseases and conditions, such as depression and alcohol abuse, probably due to the modulatory effects of sexual hormones^36,37^. Estradiol may increase the activity of ceramide synthases CerS4 and CerS5, hence enhancing S-ASM activity. However, additional research is required to elucidate these associations.

The short-/medium-/long-term effects of high levels of S-ASM in obese children has never been investigated to date. In this study, we compared erythrocyte morphological modifications in obese pediatric patients expressing high levels of S-ASM to that of healthy controls and observed no morphological differences between the two populations. This suggests that high levels of S-ASM are not associated with erythrocyte morphology in the short-term. Considering that an obese child will most likely become an obese adult^1^, it will be important to evaluate the impact of persistently elevated S-ASM values over the years on the development of erythrocyte morphological modifications and obesity-related complications.

The findings of the observational study were confirmed and further extended by our pharmacovigilance analysis: we found that children in the FAERS database who received FIASMA had a significantly lower frequency of weight gain reports than patients who took any other NON-FIASMA agents (aOR [95% CI] = 0.055 [0.0.038; 0.076]; p < 0.001]), warranting further investigation.

From a multidisciplinary point of view, this approach to the spontaneous reporting system database expands the range of potential innovative uses of pharmacovigilance data sets and offers methodologies for supporting research in the fields of clinical and basic pharmacology. The main limitations of studies based on spontaneous reporting system data include: (i) the under-reporting of adverse drug reactions (ADRs) (it is plausible to assume that expected and non-clinically relevant ADRs are more under-reported); (ii) the potential suboptimal level of data quality (including missing data); and (iii) the fact that the statistical method applied to this database (the disproportionality analysis) does not prove a certain causal relationship between the drug use and the reported ADR. Despite these inherent limitations, our findings may not necessarily be biased since (i) quantifying the reporting activity for weight gain after antipsychotic-based therapy was not our primary objective (the under-reporting was irrelevant), (ii) we only included reports with sufficient data to support a good quality statistical analysis (e.g., cases with missing data on age and sex were excluded from the analysis), (iii) we performed a disproportionality analysis for an expected ADR, given that we were not interested in highlighting any type of safety alert.

Since our primary outcome was based on subjective reporting rather than measurements, a further limitation of the pharmacovigilance analysis is the absence of a clear definition of weight changes. However, our aim was not to specifically investigate the extent and type of anthropometric variations, but to detect cases reporting increased body weight, increased waist circumference, and a high body mass index (we used a previously validated “Body changes”)^47^ following the use of antidepressant and antipsychotic drugs. It is also worth mentioning that FIASMA and non-FIASMA groups were very unbalanced numerically. Lastly, since data on therapeutic indications for the drugs presented are not always consistently reported in the database, we were unable to eliminate patients with genetic syndromes or other conditions that may contribute to obesity. However, when accounting for potential biases, we included concomitant drugs possibly associated with clinically significant weight gain as a potential confounding factor and used this information to adjust the ROR values (aROR; 95% CI). On the other hand, the major strengths of our pharmacovigilance analysis are the high number of reports, our efforts to minimize and adjust for known confounders, and the innovative approach we used to support research in the fields of clinical and basic pharmacology.

Our observational study has certain limitations. To begin with, the population we analyzed was relatively small in size. Furthermore, the absence of a complete metabolic profile prevented us from correlating S-ASM activity with the lipid profile and insulin-resistance indexes.

In conclusion, our study showed that S-ASM levels are higher in children and adolescents with essential obesity compared to those with normal weight. This was further confirmed by the positive correlation between S-ASM activity and standardised BMI. The results of the pharmacovigilance analysis indicate that S-ASM may serve as a therapeutic target for the treatment of obesity.

Future research is needed to corroborate our findings in a larger sample of obese children and to explore new associations, such as the relationship between S-ASM and the medium-/long-term implications of long-standing obesity. This pilot study could serve as the basis for future research in this interesting field.

## Material and methods

Caucasian children and adolescents aged 6 to 16 years, who were followed at the Obesity Outpatient Clinic of the V. Buzzi Children's Hospital in Milan (Italy), were recruited from November 1st, 2019 to March 30th, 2021. Obesity was defined as a BMI z-score ≥ 2 in accordance with WHO growth charts^38^. Children and adolescents with other types of obesity, systemic diseases, infections, and/or those on medications were excluded.

Caucasian, healthy, gender- and age-matched controls with a BMI z-score between –1.99 DS and + 0.99 DS according to the WHO chart were recruited as a control group using the same exclusion criteria.

When referred to the clinic, each patient and healthy control underwent a medical evaluation that included anthropometric measurements (see below), blood pressure measurement, and pubertal stage^39^. Tanner stages 1 and 2 were combined as prepubertal/early pubertal development, stages 3 and 4 as pubertal development, and stages 5 as postpubertal development. When possible, control subjects were matched by pubertal stage.

Each patient was requested to undergo the blood tests outlined below within 7 days from the medical visit. All procedures, which were performed between 8:00 a.m. and 9:00 a.m., were provided free of charge by the National Health System, as per institutional policy.

All subjects or their parents gave their written informed consent to participate in the study.

The study was approved by the Ethics Committee of the ASST-FBF-Sacco, Milan, Italy (protocol number 20118), and was conducted in accordance with the Declaration of Helsinki.

### Anthropometric measurements and blood pressure

Weight was measured using a medically-approved scale (SECA, Hamburg, Germany). The children’s height was measured using a medically-approved stadiometer (SECA, Hamburg, Germany). The waist circumference (WC) was measured twice to the nearest 0.1 cm using inextensible anthropometric tape parallel to the floor. At the end of normal expiration^40^, WC was measured midway between the lowest border of the rib cage and the upper border of the iliac crest.

Peripheral brachial blood pressure (BP) was measured three times by a trained observer using an aneroid sphygmomanometer (TEMA, Model Certus, Italy) after the patient sat still for at least 5 min. Hypertension was defined as BP ≥ 95th percentile for age, sex, and height^41^.

### Blood tests

Blood samples were obtained after a 10-h overnight fast to measure plasma glucose, total cholesterol (TC), high-density lipoprotein cholesterol (HDL), low-density lipoprotein cholesterol (LDL), triglyceride levels (TG), and alanine aminotransferase (ALT).

We used standard cut-off values for total cholesterol (hypercholesterolemia ≥ 200 mg/dl), HDL cholesterol (hypo-HDL < 40 mg/dl, < 50 mg/dl for females > 16 years), LDL cholesterol (hyper-LDL ≥ 130 mg/dl), and triglycerides (hyperTG ≥ 150 mg/dl)^42^.

### Determination of S-ASM activity in blood serum

S-ASM activity in patient serum was quantified by measuring the conversion of sphingomyelin to phosphorylcholine using a modified colorimetric assay according to the manufacturer’s instructions (Biovision Acid Sphingomyelinase Activity Colorimetric Assay Kit). Briefly, serum vials were centrifuged at room temperature (2000×*g* for 10 min), and serum was aliquoted and stored at − 80 °C for future S-ASM activity assays. S-ASM levels were assayed in 96 well plates in an acid sample buffer (pH 5) containing Zn^2+^ (500 µM ZnCl_2_). In order to check the technical and biological variance between the different samples, absorbance was measured in duplicate using a microplate spectrophotometer (OD 570 nm). Each sample’s activity (pmol/ml/h) was calculated by generating a standard curve. This was achieved by measuring the absorbance of known choline concentrations and determining the amount of product generated in samples with unknown activity from the absorbance value by reading off the intercept on the x-axis.

### Transmission electron microscopy (TEM)

For ultrastructure analysis, erythrocytes donated by all the obese pediatric patients and healthy controls were fixed at 4 °C for 2 h in 2.5% glutaraldehyde in 0.1 M cacodylate buffer (pH 7.4). After postfixation with 1% osmium tetroxide at 0 °C for 30 min, cells were stained with 2% aqueous uranyl acetate, dehydrated with ascending concentrations of acetone at 4 °C, and embedded in Epon-Araldite resin.

Red blood cell samples from 5 obese patients, to be analyzed by TEM, were subsequently screened based on the elevated levels of S-ASM activity and compared to an equivalent number of matched healthy subjects.

Ultrathin sections, obtained with a Leica Supernova ultramicrotome (Reichert Ultracut E and UC7, Leica Microsystems, Wetzlar, Germany), were stained with lead citrate and observed with a Zeiss EM10 electron microscope (Carl Zeiss, Oberkochen, Germany).

### Pharmacovigilance study

#### Data source and data processing

Data were obtained from the FAERS, a spontaneous reporting system database. The number of safety reports sent to the Food and Drug Administration (FDA) is continuously expanding (the database receives millions of reports per year), and the database is largely used to detect novel drug-related safety events to identify possible mechanisms of adverse events, to explore potential drug-drug interactions related to adverse events, and to identify promising new concomitant uses of drugs^43,44^. Adverse events are reported in the FAERS using the preferred terms from the Medical Dictionary for Regulatory Activities (MedDRA®). Adverse events recorded in the FAERS were downloaded from the official FDA website^45^. The database consists of seven data sets, namely patient demographic and administrative information (file descriptor DEMO), drug and biologic information (DRUG), adverse events (REAC), patient outcomes (OUTC), report sources (RPSR), drug therapy start and end dates (THER), and indications for use/diagnosis (INDI). Each of these seven data sets was assigned a unique identification number, and a relational database was then constructed. Data extraction was restricted to reports with values for age and gender. As previously described, duplicate records were detected and deleted accordingly^46^. Our base cohort, extracted from the FAERS database in the period covering the first quarter of 2010 and the second quarter of 2019, included all adverse events involving children aged between 5–17 years and at least one antidepressant/antipsychotic drug (Anatomical Therapeutic Chemical [ATC] code N05A or N06A) reported as suspect or interacting. Drugs were then separated into two groups based on their ASM activity. The list of FIASMA class antidepressant and antipsychotic agents included in the analysis is provided in Supplementary Table 1. Cases that reported a concomitant use of FIASMA and NON-FIASMA agents were excluded from the analyses.

### Definition of cases and case-non-case study

This study was designed as a nested case-non-case study, in which cases and non-cases are drawn from the pediatric population treated with at least one antidepressant/antipsychotic drug reported as suspect or interacting in the occurrence of any type of ADR. Our analyses were based on the MedDRA Preferred Terms describing the adverse events of interest, namely “weight-related event” and “obesity.” As recently described in a previous study^47^, we created a customized list of weight-related event terms for data mining by combining different Preferred Terms containing a range of Lowest Level Terms (LLTs) reflecting the same medical concept expressed by synonyms and lexical variants^48^ (Supplementary Table 2). Within this selected population, cases were defined as patients who developed the outcome of interest, i.e., at least one LLT from our customized list (all cases reporting a decrease in weight, abnormal weight loss, and being underweight were excluded from the analysis); non-cases were all patients who developed other adverse events (i.e., all reports without the outcome of interest), during the same period of observation.

### Statistical analysis

#### Observational study

Descriptive statistics (means, SDs, counts, and percentages) were used to describe the study populations. Pearson’s correlation coefficient was used to investigate the correlations between continuous variables. A Wilcoxon signed-rank test for paired data^49^ was used to detect a different distribution of S-ASM between cases and matched controls. A conditional logistic regression model^50^ was utilized to estimate the odds ratio (OR) of a 10 pmol/ml/h increment in S-ASM while accounting for matching. Similarly, a univariable conditional logistic model was estimated for each subject’s characteristics, and a Wald test was applied to detect a different distribution or proportion between cases and matched controls.

As an exploratory analysis, the unpaired Wilcoxon–Mann–Whitney exact test^49^ or the Fisher–Freeman–Halton exact test^51^, as applicable, were applied to detect a different distribution of characteristics between subjects categorized into high or low S-ASM expression levels, using the 3rd quartile of the S-ASM distribution as the cut-point. Variables with significant differences between the levels of the dichotomous form of S-ASM were first tested using a univariable regression model and then chosen as covariates of a multivariable logistic model.

A two-sided p-value ≤ 0.05 was deemed to be statistically significant, as were confidence intervals at 95% (95% CI). All analyses were performed using version 4.1.1. of the R open-source software (http://www.R-project.org).

### Pharmacovigilance study

A descriptive analysis was performed for cases and non-cases in terms of age, gender, the use of concomitant medications associated with body changes, and reporting type.

The reporting odds ratio (ROR) was employed to identify signs of disproportionate reporting for adverse events related to body changes in association with FIASMA/NON FIASMA agents. This estimates the frequency of an event of interest with the tested drugs relative to the other drugs.

Signals of disproportionate reporting were detected when the number of reports was higher than three and the ROR—95% CI was greater than one. In addition to the crude ROR (cROR), the adjusted ROR (aROR) was calculated using multivariate logistic regression adjusted for potential confounding factors, including age and concomitant treatments associated with body changes (Supplementary Table 3 reports the list of drugs associated with a significant weight gain). The cROR and aROR were calculated to compare the risk of body changes between FIASMA and NON FIASMA agents.

Given the high potential risk of gender as a confounding factor, we stratified the analysis to mitigate this covariate’s effect. All analyses were performed using unique case counts. Data reading, filtering, processing, and statistical analysis were performed by means of RStudio.

## Supplementary Information

Supplementary Information.

## Data Availability

All data generated and analysed during this study are included in this published article.
