# Supplementary material for: Increased acid sphingomyelinase levels in pediatric patients with obesity
Source: Sci Rep. 2022 Jun 29;12:10996. doi: 10.1038/s41598-022-14687-9 (PMC9243121; doi:10.1038/s41598-022-14687-9)

**Supplementary Table 1**: List of antidepressive and antipsychotic agents belonging to the FIASMA class included in the pharmacovigilance analysis.

| **Drug** | **ATC Code** |
| --- | --- |
| Amitriptyline | N06AA09 |
| Chlorpromazine | N05AA01 |
| Chlorprothixene | N05AF03 |
| Clomipramine | N06AA04 |
| Desipramine | N06AA01 |
| Doxepin | N06AA12 |
| Fluoxetine | N06AB03 |
| Flupentixol | N05AF01 |
| Fluphenazine | N05AB02 |
| Fluvoxamine | N06AB08 |
| Imipramine | N06AA02 |
| Lofepramine | N06AA07 |
| Maprotiline | N06AA21 |
| Nortriptyline | N06AA10 |
| Paroxetine | N06AB05 |
| Penfluridol | N05AG03 |
| Perphenazine | N05AB03 |
| Pimozide | N05AG02 |
| Promazine | N05AA03 |
| Protriptyline | N06AA11 |
| Sertindole | N05AE03 |
| Sertraline | N06AB06 |
| Thioridazine | N05AC02 |
| Trifluoperazine | N05AB06 |
| Triflupromazine | N05AA05 |
| Trimipramine | N06AA06 |

Kornhuber, J., Tripal, P., Gulbins, E., & Muehlbacher, M. (2013). Functional inhibitors of acid sphingomyelinase (FIASMAs). *Handbook of experimental pharmacology*, (215), 169–186 (2013)

**Supplementary Table 2:** List of Medical Dictionary for Regulatory Activities (MedDRA) Lowest-Level Terms to create the customised ‘Body-changes’ query.

| Must include: |
| --- |
| WEIGHT ABNORMAL |
| WEIGHT INCREASE |
| WEIGHT GAIN |
| HIGH WEIGHT |
| WEIGHT INCREASED |
| WEIGHT ABOVE NORMAL |
| ABNORMAL WEIGHT GAIN |
| WAIST CIRCUMFERENCE INCREASED |
| BODY MASS INDEX HIGH |
| BODY MASS INDEX ABNORMAL |
| BODY MASS INDEX INCREASED |
| OVERWEIGHT |
| OBESITY |
| GROSS OBESITY |
| CENTRAL OBESITY |
| TRUNCAL OBESITY |
| MORBID OBESITY |
| ABDOMINAL OBESITY |
| At the same time, cases MUST NOT include: |
| WEIGHT DECREASED |
| WEIGHT DECREASE |
| WEIGHT GAIN POOR |
| WEIGHT NORMAL |
| ABNORMAL LOSS OF WEIGHT |
| UNDERWEIGHT |

**Supplementary Table 3**: List of drugs associated with a significant weight gain.

| **Category** | **Subcategory** | **Drug related** |
| --- | --- | --- |
| Corticosteroid | Mineralocorticoid | ALDOSTERONE |
|  |  | DESOXYCORTONE  FLUDROCORTISONE |
|  | Glucocorticoid | BETAMETHASONE |
|  |  | CLOPREDNOL |
|  |  | CORTISONE |
|  |  | CORTIVAZOL |
|  |  | DEFLAZACORT |
|  |  | DEXAMETHASONE |
|  |  | FLUOCORTOLONE  HYDROCORTISONE |
|  |  | MEPREDNISONE |
|  |  | METHYLPREDNISOLONE |
|  |  | PARAMETHASONE |
|  |  | PREDNISOLONE |
|  |  | PREDNISONE |
|  |  | PREDNYLIDENE |
|  |  | RIMEXOLONE  TRIAMCINOLONE |
| Antidiabetic agent | Insulin | INSULIN |
|  | Sulfonylurea | ACETOHEXAMIDE |
|  |  | CARBUTAMIDE |
|  |  | CHLORPROPAMIDE |
|  |  | GLIBENCLAMIDE |
|  |  | GLIBORNURIDE |
|  |  | GLICLAZIDE |
|  |  | GLIMEPIRIDE  GLIPIZIDE |
|  |  | GLIQUIDONE |
|  |  | GLISOXEPIDE |
|  |  | METAHEXAMIDE |
|  |  | TOLAZAMIDE |
|  |  | TOLBUTAMIDE |
|  | Thiazolidinedione | PIOGLITAZONE |
|  |  | ROSIGLITAZONE  TROGLITAZONE |
| Antihypertensive treatment | Beta-blocker | ATENOLOL |

|  |  | METOPROLOL  PROPRANOLOL |
| --- | --- | --- |
|  | Thiazide and thiazide-like diuretic | CHLOROTHIAZIDE |
|  |  | CHLORTALIDONE |
|  |  | DIAZOXIDE |
|  |  | HYDROCHLOROTHIAZIDE |
|  |  | INDAPAMIDE  METHYLCLOTHIAZIDE |
|  |  | METOLAZONE |
| Psychotropic medication | Lithium | LITHIUM |
|  | Second generation antipsychotic | AMISULPRIDE |
|  |  | ARIPIPRAZOLE |
|  |  | CLOZAPINE |
|  |  | ILOPERIDONE |
|  |  | OLANZAPINE  PALIPERIDONE |
|  |  | QUETIAPINE |
|  |  | RISPERIDONE |
|  |  | ZIPRASIDONE |
|  | Antiepileptic drug | CARBAMAZEPINE |
|  |  | GABAPENTIN |
|  |  | PREGABALIN |
|  |  | VALPROIC ACID |
| Antibiotic | Quinolone | GATIFLOXACIN  LEVOFLOXACIN |
| Calcineurin inhibitor |  | CICLOSPORIN |
|  |  | SIROLIMUS |
|  |  | TACROLIMUS |
| Protease inhibitor |  | ATAZANAVIR |
|  |  | DARUNAVIR |
|  |  | FOSAMPRENAVIR |
|  |  | INDINAVIR |
|  |  | NELFINAVIR  RITONAVIR |
|  |  | SAQUINAVIR |
|  |  | TIPRANAVIR |

**Supplementary Figure**: Predicted log odds for each variable included in multivariable logistic regression model with High- vs Low- S-ASM as binary outcome.


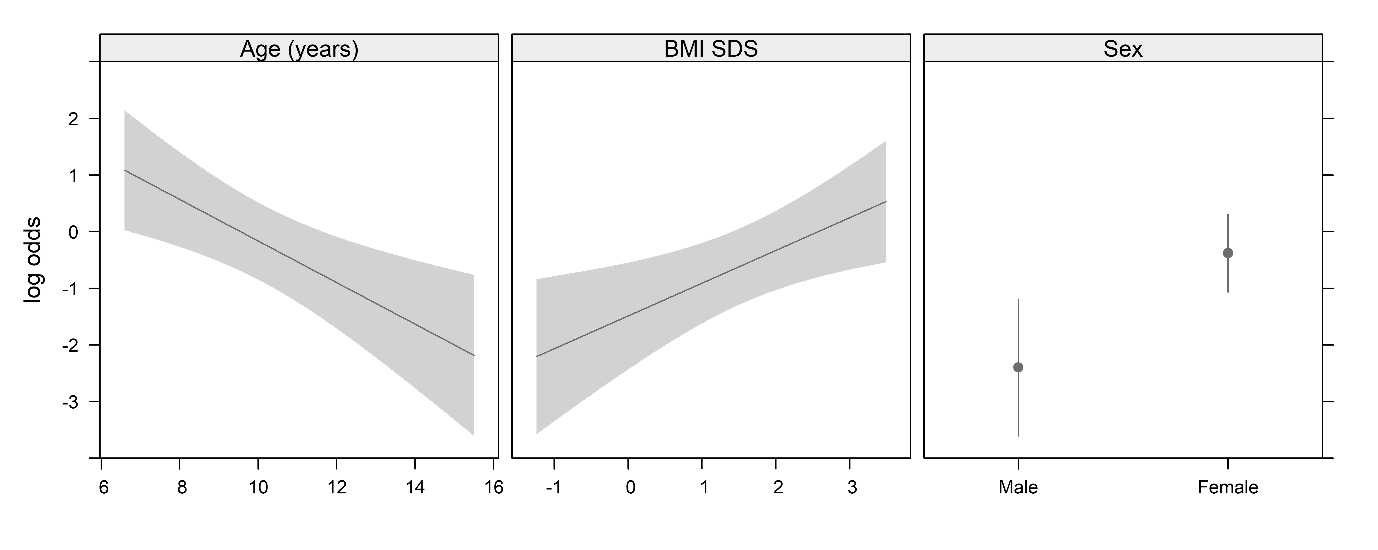

Supplement: Supplementary file 1 — Supplementary Information. [file 41598_2022_14687_MOESM1_ESM.docx]
